# Prognostic value of ubiquitin E2 UBE2W and its correlation with tumor-infiltrating immune cells in breast cancer

**DOI:** 10.1186/s12885-021-08234-4

**Published:** 2021-04-30

**Authors:** Yan Yuan, Wei-Wei Xiao, Wei-Hao Xie, Rong-Zhen Li, Yuan-Hong Gao

**Affiliations:** 1 State Key laboratory of Oncology in South China, Collaborative innovation Center for cancer Medicine, Guangzhou, P. R. China; 2 Department of Radiation Oncology, Sun Yat-sen University Cancer Center, Guangzhou, P. R. China

**Keywords:** UBE2W, Breast cancer, Prognosis, Tumor immune environment, Endocrine therapy resistance

## Abstract

**Background:**

Ubiquitin-conjugating enzyme E2W (*UBE2W*) is a protein-coding gene that has an important role in ubiquitination and may be vital in the repair of DNA damage. However, studies on the prognostic value of UBE2W and its correlation with tumor-infiltrating immune cells in multiple cancers have not been addressed.

**Methods:**

We investigated UBE2W expression in the Oncomine database, the Tumor Immune Estimation Resource (TIMER), TNMplot database. Then, the clinical prognostic value of UBE2W was analyzed via online public databases. Meanwhile, we explored the correlation between UBE2W and DNA repair associate genes expression and DNA methyltransferase expression by TIMER and Gene Expression Profiling Interactive Analysis (GEPIA). By using the same method, the correlation between UBE2W and tumor-infiltrating immune cells was explored. Genomic Profiles of UBE2W in breast cancer (BRCA) were accessed in cBioPortal (v3.5.0). Functional proteins associated network was analyzed by STRING database (v11.0).

**Results:**

UBE2W was abnormally expressed and significantly correlated with mismatch repair (MMR) gene mutation levels, DNA methyltransferase, and BRCA1/2 expression in breast cancer. High expression of UBE2W may promote the tumor immunosuppression and metastasis, induce endocrine therapy resistance and deteriorate outcomes of patients with breast cancer. These findings suggest that UBE2W could be a potential biomarker of prognosis and tumor-infiltrating immune cells. Besides, RBX1 may be a new E3 that was regulated by UBE2W.

**Conclusions:**

Ubiquitin E2 UBE2W is a potential prognostic biomarker and is correlated with immune infiltration in BRCA.

**Supplementary Information:**

The online version contains supplementary material available at 10.1186/s12885-021-08234-4.

## Introduction

Cancer is a major public health problem with increasing rapid incidence and mortality in the worldwide [1]. Over the last few decades, the various molecular mechanism of carcinogenesis has been identified, including gene mutation and epigenetic modification [2]. As an important post-translational modification, ubiquitin could participate in the oncogenesis and tumor resistance through protein degradation pathways [3].

Ubiquitin–proteasome system (UPS) mediated 80% ~ 85% protein degradation in eukaryotes [4]. The system regulates a variety of cellular activities including cell cycle, apoptosis, transcriptional regulation, DNA repair, and immune response [5, 6]. Recent findings indicate that UPS plays a key role in the carcinogenesis and progression of different types of tumors [7, 8]. The natural corollary of the above is the prediction that UPS components may be major contributory factors to human cancer. Ubiquitin-conjugating enzyme E2W (UBE2W) is an E2 ubiquitin-conjugating enzyme with direct protein N-terminal monoubiquitylation activity [9]. UBE2W could participate in the regulation of DNA repair via different pathways [10, 11]. A few recent studies disclosed that loss of ubiquitin E2 UBE2W rescues hypersensitivity of Rnf4 mutant cells to DNA damage [12]. Moreover, UBE2W was confirmed directly to participate in the ubiquitination of BRCA1/BRAD1 [13]. However, the biology and potential functions of UBE2W in tumorigenesis and mechanism are poorly understood. We speculated that UBE2W might be involved in tumorigenesis by ubiquitinating substrate N-terminal.

Additionally, UPS exerts the ability to regulate protein stability and function which is critical for the regulation of immunity [14]. For instance, ubiquitin-protein ligase E3 component N-recognin 5 (UBR5) could promote tumor proliferation by inhibiting the immune response by decreasing the CD8+ T cell [15]. Ubiquitin-conjugating enzyme UBE2D3 was also reported to regulate immunity via the NF-kB signaling pathway [16]. Thus, we speculated UBE2W may be involved in the tumor immune microenvironment.

In the present study, we primarily focus on the association between UBE2W expression and patients’ prognosis in different cancer types. The correlation between UBE2W expression and the immune environment was also explored.

## Methods

### Gene expression analysis

UBE2W mRNA expression levels were analyzed through the Oncomine database (https://www.oncomine.org) [17]. The threshold settings were as follows: gene ranking of the top 10%, fold change of 2.0, and *P*-value of 0.01. The Tumor Immune Estimation Resource (TIMER) database (https://timer.comp-genomics) and TNMplot database (http://www.tnmplot.com) were used to verify the expression of UBE2W in various cancers [18–21]. TNMplot database (http://www.tnmplot.com) with RNA-sequence data and gene-chip data were used to explore the UBE2W mRNA expression in tumor, normal, and metastatic tissues. Immunohistochemical staining pictures of UBE2W protein expression were obtained from the Human Protein Atlas (HPA) database (https://www.proteinatlas.org/) [22].

### Survival and prognosis analysis

The association of UBE2W with prognosis in different tumors via PrognoScan database (http://dna.bio.kyutech.ac.jp/PrognoScan) and Kaplan-Meier-plotter (KM plotter) database (http://kmplot.com/analysis/) [23, 24]. Subgroups survival analysis was provided in Kaplan-Meier survival plots. *P*-value < 0.05 was considered statistically significant. The ROC plotter (http://www.rocplot.org) was used to predict the UBE2W expression and response to therapy in 3104 BRCA patients [25].

### Correlation analysis between UBE2W expression and immune characteristics

The correlation between the expression of UBE2W with the abundances of six tumor-infiltrating immune cells (TIICs) (B cells, CD4+ T cells, CD8+ T cells, macrophages, neutrophils, and dendritic cells) was explored via the TIMER database. Sixty-six related gene markers of TIICs derived from the CellMarker database (http://biocc.hrbmu.edu.cn/CellMarker/) were used for the analysis [26]. Gene Expression Profiling Interactive Analysis (GEPIA) (http://gepia.cancer-pku.cn/index.html) was used to validate the gene correlation analysis in TIMER [27].

### Correlation analysis between UBE2W and BRCA1/2 genes, MMR genes, DNA methyltransferase analysis

Both the TIMER database and GEPIA database was used to evaluate the relationship between UBE2W expression and DNA repair gene (BRCA1/2 genes, five MMR genes), DNA methyltransferase genes. MMR genes contain MLH1, MSH2, MSH6, PMS2, and EPCAM. Meanwhile, DNA methyltransferase genes include DNMT1, DNMT2, DNMT3A, and DNMT3B.

### Genomic analysis

cBioPortal for Cancer Genomics (v3.5.0) (http://www.cbioportal.org/) is a powerful platform that contains DNA copy number data, mRNA and microRNA expression data, non-synonymous mutations, protein level, and clinical data of various tumors [28, 29]. The genomics features of UBE2W on a specific cancer type were analyzed via the cBioPortal database. Besides, the association between the CNV alteration of UBE2W with survival was also displayed.

### Functional protein association network analysis

STRING database (https://string-db.org/) is a powerful tool for the analysis of protein interactions involving 2031 species, 9,643,763 proteins, and 1,380,838,440 interactions [30]. The co-expression protein of UBE2W was obtained from the cBioPortal database analysis. The proteins network was established via the STRING database.

### Statistical analysis

Analysis of UBE2W expression differences based on different databases, so the test methods are different. In the Oncomine database, Student’s t-test was used to analyze the two-class differential expression. The statistical significance was computed by the Wilcoxon test in the TIMER database. While the significant differences in the TNM plot database were analyzed by the Mann-Whitney U test. Survival analysis with *p* values was based on a log-rank test. Spearman’s correlation coefficient and the *P*-value were used to evaluate the gene correlation. P-value < 0.05 was considered statistically significant. The flow diagram is shown in Supplementary Figure 1.

## Results

### The high mRNA expression level of UBE2W in breast Cancer

**Fig. 1:**
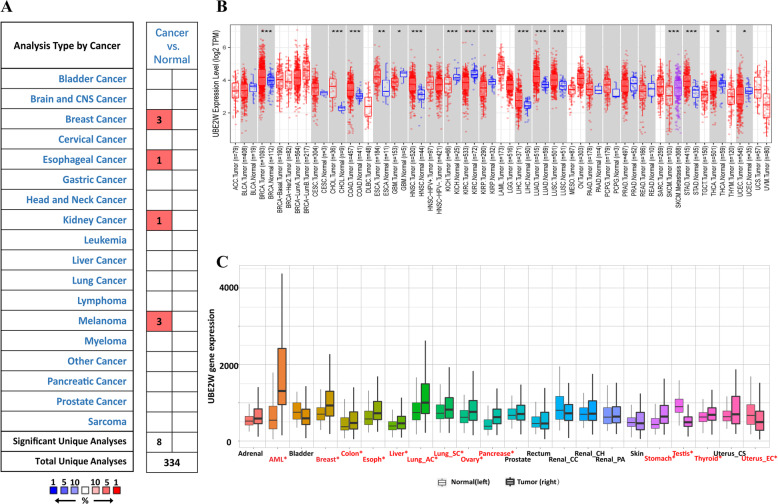
Expression of UBE2W in various human tumors. **a** Increased expression of UBE2W in different tumors compared to normal tissues in the Oncomine database. **b** UBE2W expression of different tumor types from the TCGA database was investigated by TIMER (*P < 0.05, **P < 0.01, ***P < 0.001). **c** UBE2W was abnormally expressed in pan cancers by TNMplot. Significant differences by Mann-Whitney U test are marked with red color. Abbreviations: ACC: adrenocortical carcinoma; BLCA: bladder urothelial carcinoma; BRCA: breast invasive carcinoma; CESC: cervical squamous cell carcinoma; CHOL: cholangiocarcinoma; COAD: colon adenocarcinoma; DLBC: lymphoid neoplasm diffuse large B cell lymphoma; ESCA: esophageal carcinoma; GBM: glioblastoma multiforme; LGG: brain lower grade glioma; HNSC: head and neck squamous cell carcinoma; KICH: kidney chromophobe; KIRC: kidney renal clear cell carcinoma; KIRP: kidney renal papillary cell carcinoma; LAML: acute myeloid leukemia; LIHC: liver hepatocellular carcinoma; LUAD: lung adenocarcinoma; LUSC: lung squamous cell carcinoma; MESO: mesothelioma; OV: ovarian serous cystadenocarcinoma; PAAD: pancreatic adenocarcinoma; PCPG: pheochromocytoma and paraganglioma; PRAD: prostate adenocarcinoma; READ: rectum adenocarcinoma; SARC: sarcoma; SKCM: skin cutaneous melanoma; STAD: stomach adenocarcinoma; TGCT: testicular germ cell tumors; THCA: thyroid carcinoma; THYM: thymoma; UCEC: uterine corpus endometrial carcinoma; UCS: uterine carcinosarcoma; and UVM: uveal melanoma; AML: ; Lung_AC: lung adenocarcinoma; Lung_SC: lung squamous cell carcinoma; Renal_CC: renal clear cell carcinoma; Renal_CH: renal chromophobe cell carcinoma; Renal_PA: renal papillary cell carcinoma; Uterus_CS: uterine carcinosarcoma; Uterus_EC: uterine corpus endometrial carcinoma

First, we analyze the expression level of UBE2W via the Oncomine database (Fig. 1a, *p*-value = 0.05, fold change = 2). Several datasets showed high expression levels of UBE2W was significantly in cancer samples verse in normal samples, including breast cancer (BRCA), head and neck cancers (HNSC), lymphoma, colorectal cancer, melanoma (Supplementary Table 1). Next, we further investigate the mRNA expression levels of UBE2W in The Cancer Genome Atlas (TCGA) based on TIMER database (Fig. 1b). The expression of UBE2W was absolutely higher in breast cancer than normal tissues similarly. The higher mRNA expression level of UBE2W was also detected in the cholangiocarcinoma (CHOL), colon adenocarcinoma (COAD), esophageal carcinoma (ESCA), head and neck tumor (HNSC), liver hepatocellular carcinoma (LIHC), lung adenocarcinoma (LUAD), lung squamous cell carcinoma (LUSC), stomach adenocarcinoma (STAD) than in the normal tissues. In addition, TNMplot database with RNA-seq data from TCGA, Genotype-Tissue Expression GTEX, Therapeutically Applicable Research to Generate Effective Treatments (TARGET) were used to verify the expression of UBE2W (Fig. 1c). Taken together, these results strongly demonstrated the UBE2W gene was abnormally regulated in multiple cancers than matched normal tissues, especially in breast cancer.

### High UBE2W expression correlated with poor prognosis in breast Cancer

**Fig. 2:**
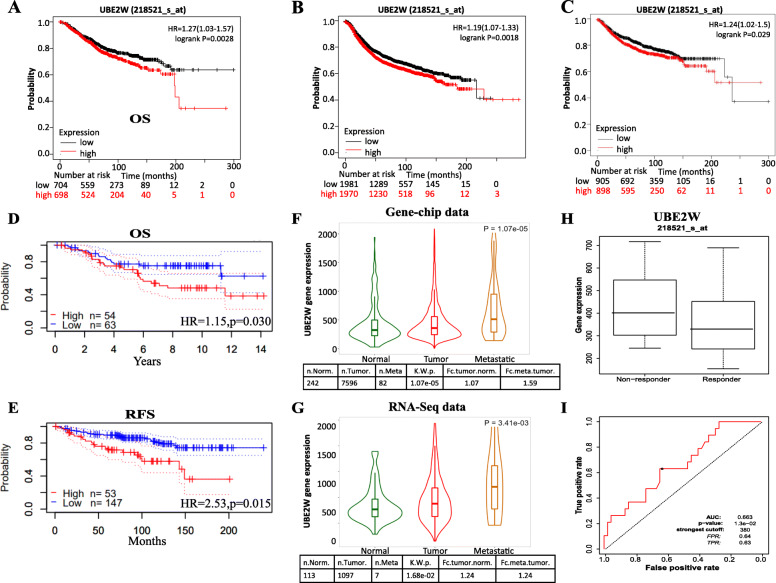
Survival analysis and predictive value analysis of UBE2W in public databases. **a**-**c** Overall survival (OS), Relapse free survival (RFS), and Distant metastasis-free survival curves of BRCA by different expression levels of UBE2W in the K-M plotter database. **d**-**e** OS and RFS curves of BRCA by different expression levels of UBE2W in the PrognoScan database. **f** Gene chip data of UBE2W expression in normal, tumor, metastatic tissues. **g** RNA-seq data of UBE2W expression in normal, tumor, metastatic tissues. **h** UBE2W expression between responders and non-responders in endocrine therapy of BRCA. **i** The ROC plot of UBE2W expression in predicting the effect of endocrine therapy in BRCA. Abbreviations: OS: overall survival; RFS: relapse-free survival; DMFS: distant metastasis free survival

PrognoScan database was used to analyze the correlation of UBE2W expression with prognosis. Six out of eleven cancers showed a potential relevance between the UBE2W expression with prognosis (Supplementary Table 2). A higher expression level of UBE2W indicated a bad overall survival (OS) (Fig. 2d) and relapse free survival (RFS) (Fig. 2e) in breast cancer. The same trend was also seen in melanoma, meningioma, colorectal cancer (Supplementary Figure 2A-D). While, higher UBE2W expression was correlated to the better survival prognosis among the other three solid cancers (astrocytoma, glioma, lung cancer) (Supplementary Figure 2E-I).

Next, we further analyze the prognostic value of UBE2W in the KM plotter database. Notably, higher UBE2W expression was still correlated to the bad prognosis in breast cancer not only in OS (Fig. 2a) and RFS (Fig. 2b) but also in distant-metastasis free survival (DMFS) (Fig. 2c, HR = 1.24, *p* = 0.029) (*p*-value < 0.05). Besides, it is also significantly related to the shorter survival in ovarian cancer (Supplementary Figure 2 J), LIHC (Supplementary Figure 2 K), rectum adenocarcinoma (Supplementary Figure 2 L), esophageal adenocarcinoma, UCEC (data has not shown). Consistent with the results of PrognoScan analysis, a higher expression level of UBE2W was correlated with better prognostic in lung cancer (Supplementary Figure 2D), gastric cancer, KIRC, and esophageal squamous cell carcinoma. In general, UBE2W has an impotent prognostic value in various tumors, especially in breast cancer. And UBE2W may play a crucial role in the progress of breast cancer.

### High expression UBE2W deteriorates the outcomes of BRCA by promoting metastasis and endocrine resistance

Besides, we further explored the mechanism of why the UBE2W expression could affect the prognosis in breast cancer. First, the difference of UBE2W expression levels in the normal, tumor, metastatic tissues was analyzed via the TNMplot database. Both gene-chip data and RNA-seq data reveal a higher expression level of UBE2W in metastatic samples than in tumors (Fig. 2f, g). Survival analysis further verified the high expression UBE2W was correlated with poor distant metastasis-free survival (Fig. 2c). Second, the correlation between the expression level of UBE2W and clinical variables was analyzed by KM plotter analysis (Table 1). Specifically, compared with low UBE2W mRNA expression level, high UBE2W mRNA expression level was related to poor prognosis only in luminal A subtype disease (OS HR = 1.43, *p* = 0.046, RFS HR = 1.34, *p* < 0.001), which was defined as human epidermal growth factor receptor (HER2) negative, progesterone receptor (PR) positive, estrogen receptor (ER) positive, Ki-67 < 14%. Then, we explored the predictive value of UBE2W expression level in response to endocrine therapy of breast cancer. By the ROC plotter analysis, the expression level of UBE2W was higher in non-responder patients than in responder patients (Fig. 2h). The expression level of UBE2W could predict the response to endocrine therapy with an AUC value of 0.663 and *p* < 0.05 (Fig. 2i). The above results indicate that high UBE2W expression could promote tumor metastasis and cause resistance to endocrine therapy in breast cancer.

**Table 1:** Correlation of UBE2W mRNA expression and clinicopathological factors in Breast cancer by Kaplan-Meier plotter database

Variables of breast cancer	Overall survival (***n*** = 1402)			Relapse free survival (***n*** = 3951)		
	N	Hazard ratio	P-value	N	Hazard ratio	P-value
ER						
Positive	548	1.58 (1.1–2.27)	**0.0117**	2061	0.96 (0.82–1.13)	0.6565
Negative	251	1.28 (0.81–2.02)	0.2954	801	0.99 (0.79–1.24)	0.9333
PR						
Positive	83	0.52 (0.13–2.11)	0.3533	589	0.789 (0.56–1.12)	0.18
Negative	89	1.12 (0.44–2.84)	0.8117	982	0.59 (0.48–0.72)	< 0.001
HER2						
Positive	129	0.84 (0.41–1.71)	0.6283	252	0.69 (0.44–1.07)	0.094
Negative	130	1.11 (0.45–2.73)	0.8279	800	0.88 (0.67–1.14)	0.324
Intrinsic subtype						
basal	241	0.96 (0.59–1.57)	0.8688	618	1.16 (0.91–1.5)	0.23
luminal A	611	**1.43 (1–2.04)**	**0.0459**	1933	**1.34 (1.13–1.59)**	**0.0009**
luminal B	433	1.02 (0.71–1.48)	0.9046	1149	1.04 (0.86–1.26)	0.7
HER2+	117	1.42 (0.74–2.72)	0.2945	251	1.18 (0.8–1.73)	0.41
Lymph node status						
Positive	313	1.13 (0.76–1.68)	0.5311	1133	1.04 (0.85–1.26)	0.7266
Negative	594	1.12 (0.77–1.63)	0.5502	2020	1.02 (0.86–1.2)	0.8565
Grade						
1	161	0.8 (0.32–1.99)	0.63	345	1.08 (0.64–1.81)	0.7806
2	387	1.16 (0.75–1.78)	0.5	901	0.98 (0.77–1.24)	0.8491
3	503	1.29 (0.93–1.8)	0.13	903	1.09 (0.87–1.35)	0.46
TP53 mutation						
Positive	111	1.81 (0.83–3.97)	0.13	188	1.56 (0.97–2.51)	0.0655
Negative	187	1.19 (0.62–2.28)	0.5973	273	0.86 (0.56–1.31)	0.4765

### Association between UBE2W expression with DNA repair and DNA methyltransferase in breast cancer

**Fig. 3:**
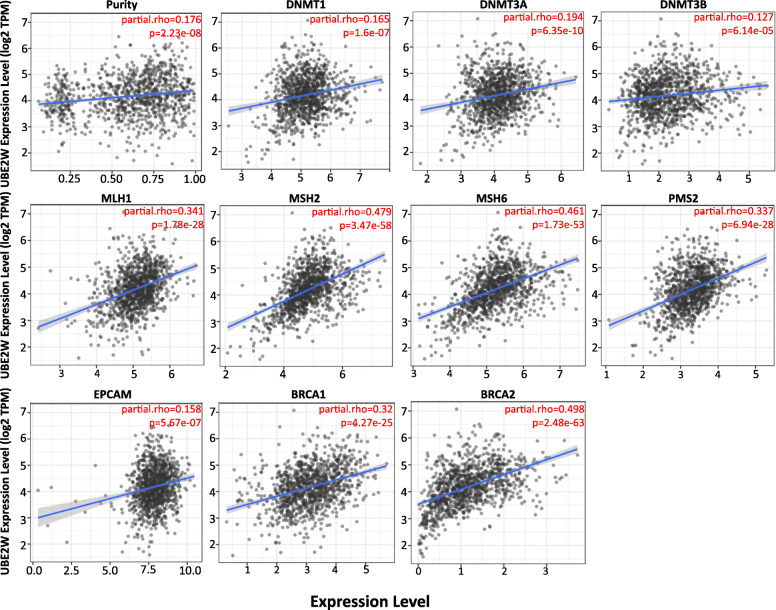
Correlation of UBE2W expression with DNA repair and DNA methyltransferase genes in BRCA

DNA mismatch repair (MMR) plays a critical role in DNA repair pathways [31]. In similar, BRCA genes contribute to DNA repair and regulate the response of the organism to DNA damage [32]. DNA methyltransferase could affect the expression of tumor-suppressing genes by the means of epigenetic modification [33]. All those genes were certified to relevant with the tamoxifen resistance and the prognosis of breast cancer [34–36]. Therefore, a correlation analysis of UBE2W expression with DNA repair genes and DNA methyltransferase genes in BRCA was pursued using the TIMER and verified via the GEPIA database. Results showed that UBE2W expression exhibited a positive relationship to those genes (DNMT1, DNMT2, DNMT3A, DNMT3B, MLH1, MSH2, MSH6, PMS2, EPCAM, BRCA1, BRCA2) in breast cancers (Fig. 3, Table 2). These results indicate that UBE2W may mediate therapy resistance by regulating DNA damage or methylation in breast cancer.

**Table 2:** Correlation of UBE2W expression and various genes in GEPIA database

	BRCA (normal)		BRCA (tumor)	
	R	P-value	R	P-value
DNMT1	0.42	4.7e−06	0.057	0.059
DNMT2	0.74	0	0.22	**1e−13**
DNMT3A	0.25	0.0089	0.13	**3.20E-05**
DNMT3B	0.38	4.5e−05	0.088	**0.0038**
EPCAM	0.24	0.011	0.17	**3.80E-08**
MLH1	0.4	1.1e−05	0.31	**0**
MSH2	0.6	2.7e−12	0.3	**0**
MSH6	0.57	5.5e−11	0.3	**0**
PMS2	0.64	3.1e−14	0.48	**0**
BRCA1	0.45	4.8e−07	0.36	**0**
BRCA2	0.42	3.1e−06	0.26	**0**

### Correlation of UBE2W expression with tumor immune microenvironment in breast cancer

**Fig. 4:**
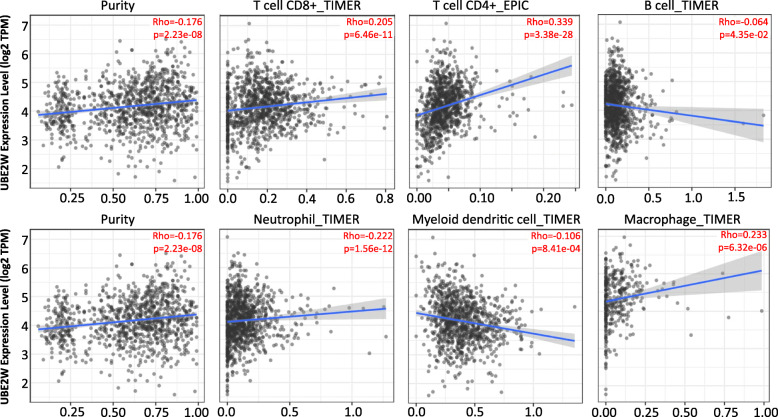
Correlation of UBE2W expression with immune infiltration level in BRCA

Antiestrogens could induce the immunosuppression in the tumor microenvironment (TME) of BRCA which caused the drug resistance of endocrine therapy [37]. Then, we explored the correlation of the UBE2W expression with the tumor-infiltrating immune cells in BRCA via TIMER analysis (Fig. 4). We observed that UBE2W expression levels were significantly positively associated with tumor purity (r = 0.176, *P* = 2.23e-08) in breast cancer. In addition, UBE2W expression was significantly positive correlated with the level of CD8+ T cells (r = 0.205, *P* = 6.46e-11), neutrophil (r = 0.222, *P* = 1.56e-12) and macrophage cells (r = 0.342, *P* = 1.27e-28). However, there were significantly negative correlation with level of CD4+ T cells (r = − 0.198, *P* = 2.95e-10) and Myeloid dendritic cells (r = − 0.106, *P* = 8.41e-04). And no significant correlation between the expression level of UBE2W with the level of B cells (r = − 0.064, *P* = 4.35e-02). These results strongly exhibited that UBE2W could recruit the immune cells in the tumor microenvironment in BRCA.

Furthermore, we analyzed the relationships between UBE2W expression with various markers of immune cells in BRCA via a public database. In the TIMER database, it was conspicuous that UBE2W expression was significantly correlated with the gene markers of monocytes/macrophages (monocytes, and M2 macrophages) than gene markers of other immune cells (Supplementary Table 3). Elevated UBE2W expression was positively related to the genes of CD206, CD163, IL10 which were the specific markers of M2 macrophages. However, in the GEPIA database, UBE2W expression was a negative correlation with the gene markers of CD8+ T cells and general T cells in BRCA tumors (Supplementary Table 4). Significant relationships were also detected in the gene markers of T cell exhaustion, monocyte with the expression level of UBE2W. In brief, those results hinted that UBE2W may play a promoting role in regulating the polarization of macrophages and decreasing the infiltration of CD8+ T cells in the TME.

### Genomic alterations and the co-expression genes of UBE2W in the BRCA

**Fig. 5:**
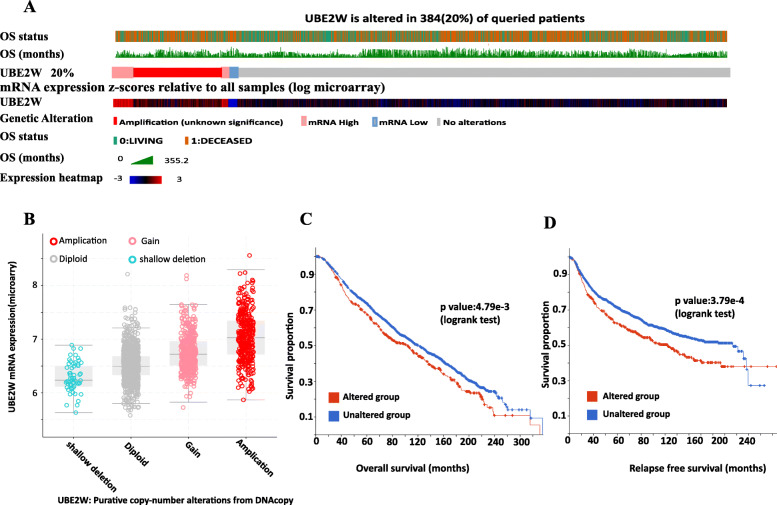
Genomic alterations of UBE2W of BRCA in cBioPortal. **a** Oncoprint of UBE2W alteration in BRCA. **b** Mutation details for UBE2W. **c**, **d** Survival analysis. Abbreviations: OS: overall survival

A cohort of 1904 breast cancer patients with complete cancer genomics data was selected from 1 of 18 studies in the cBioPortal database [38–40]. The OncoPrint shows that the UBE2W gene was altered in 20% BRCA patients and the mRNA expression level of UBE2W for all patients (Fig. 5a). We further accessed that UBE2W mRNA expression was elevated in amplified cases (Fig. 5b) and that cases with UBE2W alteration have worse overall survival (Fig. 5c) and relapse-free survival (Fig. 5d) than cases without UBE2W alteration (OS: *p* = 4.790e-3, log-rank test; RFS: *p* = 3.790e-4, log-rank test).

To find the interaction E3 ubiquitin ligase of UBE2W, we further accessed the co-expression genes of UBE2W in BRCA and performed a network analysis in the public database. Co-expression genes were obtained from the cBioPortal database (data has not shown). Then a network analysis was constructed via STRING analysis (Supplementary Figure 3B) which implied the RBX1 may be the E3 ubiquitin ligase of UBE2W. To confirm this result, we analyzed the expression level and prognosis value of the RBX1 gene in the BRCA (Supplementary Figure 4). Taken these results together, it revealed that UBE2W could affect the prognosis of BRCA through the interaction of E3 ubiquitin-protein ligase RBX1.

## Discussion

UBE2W is a protein-coding gene localized on chromosome 8q21.11 and was first reported in 2006 [41]. At present, researches on UBE2W are mainly about neurodegenerative diseases, Fanconi anemia, Huntington’s disease, and male reproductive systems [12, 42, 43]. UBE2W downregulation could promote spermatogenic cell apoptosis through activating the P53/Bcl-2/caspase 6/caspase 9 signal pathways [44]. The loss of UBE2W could sensitized cells to the DNA damage agents with the absence of Rnf4 [12]. However, the roles of UBE2W in human pan-cancer has not been identified. Whether UBE2W could be used as a biomarker is still unknown. An integrated analysis that we performed reveals that UBE2W was a potential oncogenic gene with prognostic value in BRCA, which is the first study of the UBE2W gene with cancer. According to the results from the Human Protein Atlas (HPA) database (https://www.proteinatlas.org/), UBE2W exhibits a significantly higher expression profile in PTC tissues than in normal tissues (NT) (Supplementary Figure 3A). Notably, we found higher expression of UBE2W in metastatic tissues than primary tumors were observed in BRCA. Meanwhile, the high expression level of UBE2W is significantly correlated with the non-response of endocrine therapy. We further detected that UBE2W expression was correlated with DNA repair and methylation. Immune cell infiltration levels and various immune-related markers were also employed to analyze the relationship with UBE2W in specific cancer. Also, an exploration of the genomic alteration of UBE2W was performed. Therefore, our study provides clues to shed light on the potential effects of UBE2W in cancer research.

In the present research, we verified different expression levels of UBE2W in normal and tumor from multi-omics data integration and analysis in specific cancer. Firstly, the difference mRNA expression level of UBE2W between tumor and normal was performed in various public datasets. Oncomine datasets shown UBE2W expression was upregulated in breast cancer, colorectal cancer, head and neck cancer, lung cancer. In the TIMER dataset, higher UBE2W expression was detected in BRCA, CHOL, COAD, ESCA, GBM, HNSC, LUAD, LUSC, STAD. While in the TNM plot database which contained includes 56,938 unique multilevel quality control samples, results of UBE2W expression were similar to the TIMER analysis. Three public analysis platforms confirmed the higher expression level of UBE2W in BRCA than the normal tissues. Compare the mRNA expression level of UBE2W in the metastatic tumors to the primary tumors, a rising trend was detected. Secondly, we found UBE2W exhibited a significantly higher expression profile in BRCA tumor tissues than in normal tissues. Finally, the genomic analysis revealed that higher UBE2W mRNA expression was related to the copy number amplification in BRCA. In summary, it confirms that UBE2W expression was significantly higher in tumors rather than normal tissues in BRCA. A high expression level of UBE2W may promote tumor metastasis in BRCA. Then, a consistent result regarding the prognosis of UBE2W was found in BRCA via PrognoScan and KM plotter analysis. Meanwhile, UBE2W expression could be a predictive biomarker in the response of endocrine therapy in BRCA which was confirmed by the Cox regression analysis in the KM plotter database. Moreover, UBE2W genomic alteration was also correlated with the poor prognosis survival in BRCA. In brief, those results prompt that UBE2W is a prognostic biomarker for breast cancer. Combined co-expression analysis and survival analysis, we found RBX1 was a gene co-expression with UBE2W and high RBX1 expression was correlated with the poor survival in BRCA. Previous studies pointed out that RBX1 expression could regulate the tumor suppressor degradation in liver cancer and be a prognostic factor in lung cancer [45, 46]. Therefore, we guess that RBX1 may be a new E3-ubiquitin ligase of UBE2W in BRCA.

Dysregulation of DNA damage repair (DDR) genes was reported to be a factor that promoting endocrine therapy resistance [34, 47]. Both BRCA1 and BRCA2 are well-known genes concerning homologous recombination in DDR [48]. A novel resistance mechanism with re-express BRCA1 causes the acquisition of therapy resistance [36]. And BRCA2 mutation was associated with disease progression in breast cancer [49]. While MMRs consist of multiple genes (MLH1, MSH2, MSH6, PML2, EPCAM) were demonstrated to induce endocrine therapy resistance via suppressing the CDK4 activity [34]. And the function loss of those genes brings about a mutator phenotype which causes an increasing tumor risk [50]. In this study, we found that UBE2W expression was closely related to the expression level of BRCA1/2 and 5 MMR genes in breast cancer. Previous studies have shown that DNMTs (DNMT1, DNMT3A, DNMT3B) were overexpressed in tamoxifen-resistance tumors and DNMT3B remained as an independent prognostic survival factor for BRCA [35]. In this study, UBE2W expression was positively related to the expression of DNMTs in breast cancer. Furthermore, we analyzed the correlation of UBE2W with various genes (EGFR, ERBB2, ESR1, FOXM1, GATA3, GREB1, IGF1R, NCOR1, PIK3CA, PTEN) which were reported to be a promoter of endocrine therapy resistance [51, 52]. Results showed that UBE2W expression was positively correlated to all those genes in breast cancer, especially in the luminal A subtype (Supplementary Figure 3C). All those results demonstrated that a high expression level of UBE2W may induce poor prognosis survival through promoting the endocrine therapy resistance in breast cancer, especially in the subtype of luminal A.

It was reported that antiestrogen treatment could induce immunosuppression in TME which may contribute to the endocrine therapy in BRCA [37]. In this study, we found that UBE2W expression was associated with tumor infiltration immune cells in BRCA. Interestingly, UBE2W expression was positive correlated with the expression of CD206, CD163, and IL10 in the TIMER database. While, in the GEPIA database, UBE2W was negative correlated with the immune-specific genes of CD8+ T cells. These results exhibited that UBE2W expression could promote immunosuppression in TME.

Nevertheless, our research has some limitations. Considering that our study was based on public databases, systematic bias cannot be avoided. The mechanism of UBE2W in promoting cancer progression and therapy resistance needs a more rigorous exploration. Although our results discover the correlation of UBE2W expression with TME, further efforts are required to verify whether UBE2W expression could affect the prognosis of BRCA across the tumor infiltration immune cells.

## Conclusions

In conclusion, UBE2W was an unfavorable prognostic factor and could be a predictive biomarker of endocrine therapy in BRCA. UBE2W might promote tumor metastasis and the resistance of endocrine therapy though influencing the process of DNA repair and DNA methyltransferase, inducing the tumor immunosuppression. Besides, RBX1 may be a new ubiquitinated ligase of UBE2W in BRCA.

## Supplementary Information

**Additional file 1: Supplementary Figure 1**. Flow diagram. **Supplementary Figure 2**. Impact of gene expression of UBE2W on various cancer survival in PrognoScan database. Overall survival (OS) of skin cancer(A), eye cancer (B), brain cancer (C, F, G), colorectal cancer (D), ovarian cancer (J), liver hepatocellular carcinoma (K), rectum adenocarcinoma (L), lung cancer (H). Relapse-free survival (RFS) of lung cancer (I). Disease-free survival (DFS) of colorectal cancer (E). **Supplementary Figure 3**. (A) UBE2W protein expression profile in the HPA database. (B) Network view of theUBE2W co-expression genes in breast cancer. (C) Correlation of UBE2W expression with various genes in BRCA in TIMER. Significant positive correlations by the Spearman test are marked with red color (p <0.05). No significant correlations are marked with gray color (p>0.05). **Supplementary Figure 4**. Expression and prognosis value of RBX1. (A) RBX1 expression in different cancers in TIMER (***P < 0.001). (B) RBX1 expression level in BRCA in Oncomine. (C) UBE2W expression in normal, tumor, metastatic tissues in TNMplot. (E, F) Overall survival and relapse-free survival analysis of RBX1in Kaplan-Meier Plotter. (G, H) Overall survival and relapse-free survival analysis of RBX1in PrognoScan. **Supplementary Table 1**. UBE2W expression in cancers vs normal tissue in Oncomine database (positive). **Supplementary Table 2**. Positive results associated with UBE2W expression in different cancers from the Prognoscan database. **Supplementary Table 3**. Correlation analysis between UBE2W and markers of infiltrating immune cells in TIMER. **Supplementary Table 4**. Correlation analysis between UBE2W and markers of infiltrating immune cells in GEPIA.

## Data Availability

All data are available via the corresponding author.
