# Supplementary material for: Prognostic value of ubiquitin E2 UBE2W and its correlation with tumor-infiltrating immune cells in breast cancer
Source: BMC Cancer. 2021 Apr 30;21:479. doi: 10.1186/s12885-021-08234-4 (PMC8086329; doi:10.1186/s12885-021-08234-4)
Supplement: Supplementary file 1 — Additional file 1: Supplementary Figure 1. Flow diagram. Supplementary Figure 2. Impact of gene expression of UBE2W on various cancer survival in PrognoScan database. Overall survival (OS) of skin cancer(A), eye cancer (B), brain cancer (C, F, G), colorectal cancer (D), ovarian cancer (J), liver hepatocellular carcinoma (K), rectum adenocarcinoma (L), lung cancer (H). Relapse-free survival (RFS) of lung cancer (I). Disease-free survival (DFS) of colorectal cancer (E). Supplementary Figure 3. (A) UBE2W protein expression profile in the HPA database. (B) Network view of theUBE2W co-expression genes in breast cancer. (C) Correlation of UBE2W expression with various genes in BRCA in TIMER. Significant positive correlations by the Spearman test are marked with red color (p <0.05). No significant correlations are marked with gray color (p>0.05). Supplementary Figure 4. Expression and prognosis value of RBX1. (A) RBX1 expression in different cancers in TIMER (***P < 0.001). (B) RBX1 expression level in BRCA in Oncomine. (C) UBE2W expression in normal, tumor, metastatic tissues in TNMplot. (E, F) Overall survival and relapse-free survival analysis of RBX1in Kaplan-Meier Plotter. (G, H) Overall survival and relapse-free survival analysis of RBX1in PrognoScan. Supplementary Table 1. UBE2W expression in cancers vs normal tissue in Oncomine database (positive). Supplementary Table 2. Positive results associated with UBE2W expression in different cancers from the Prognoscan database. Supplementary Table 3. Correlation analysis between UBE2W and markers of infiltrating immune cells in TIMER. Supplementary Table 4. Correlation analysis between UBE2W and markers of infiltrating immune cells in GEPIA. [file 12885_2021_8234_MOESM1_ESM.docx]

**Prognostic value of ubiquitin E2 UBE2W and its correlation with tumor-infiltrating immune cells in breast cancer**

**Supplementary information**

Supplementary Figure 1. Flow diagram.

Abbreviations: IHC: immunohistochemistry; BRCA: breast carcinoma; TME: tumor immune microenvironment; TIICs: tumor infiltrating immune cells.

**
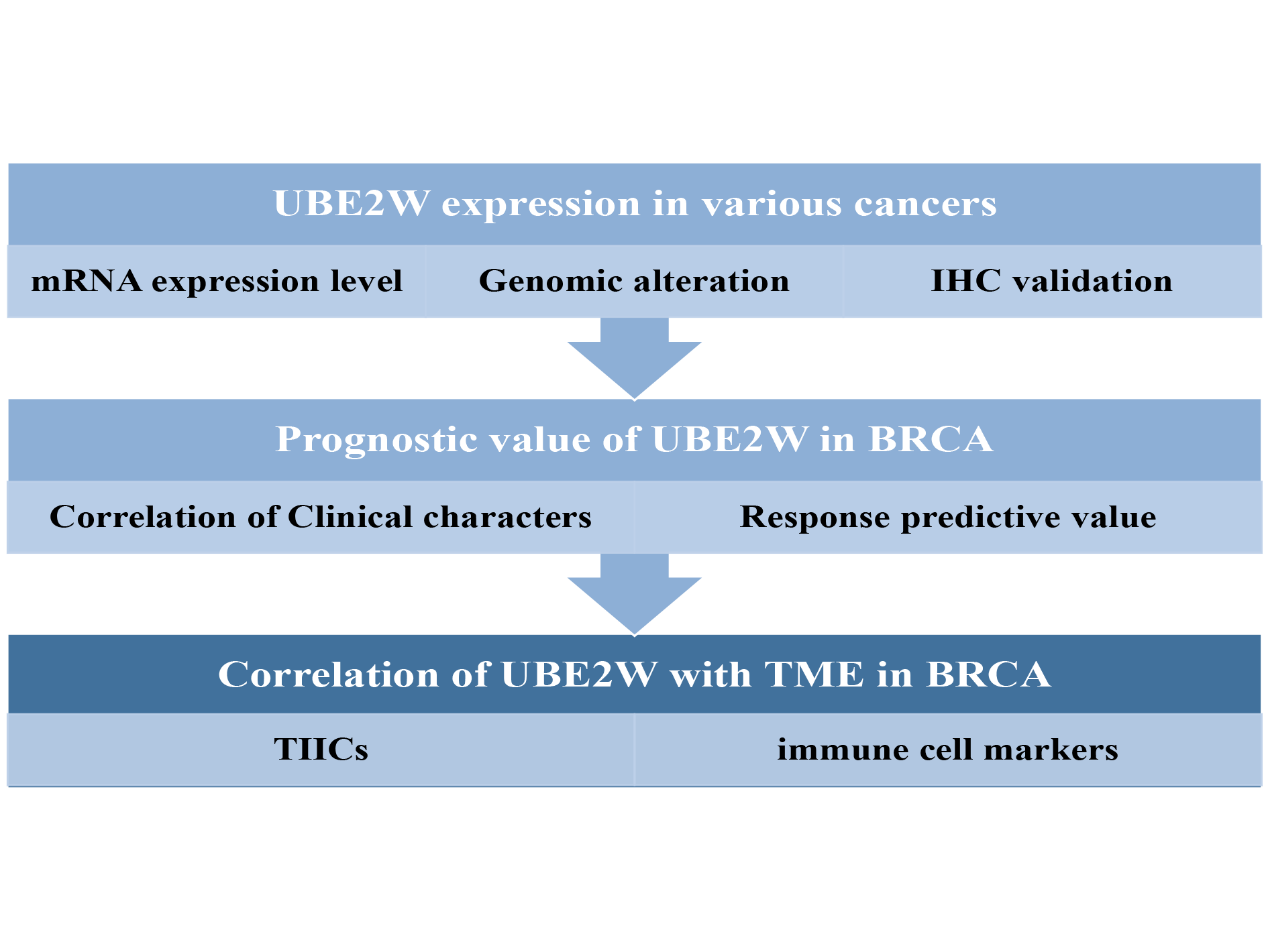
**

Supplementary Figure 2. Impact of gene expression of UBE2W on various cancer survival in PrognoScan database. Overall survival (OS) of skin cancer(A), eye cancer (B), brain cancer (C, F, G), colorectal cancer (D), ovarian cancer (J), liver hepatocellular carcinoma (K), rectum adenocarcinoma (L), lung cancer (H). Relapse-free survival (RFS) of lung cancer (I). Disease-free survival (DFS) of colorectal cancer (E).

Abbreviations: OS: overall survival; RFS: relapse-free survival; DFS: disease-free survival; HR: hazard ratio.


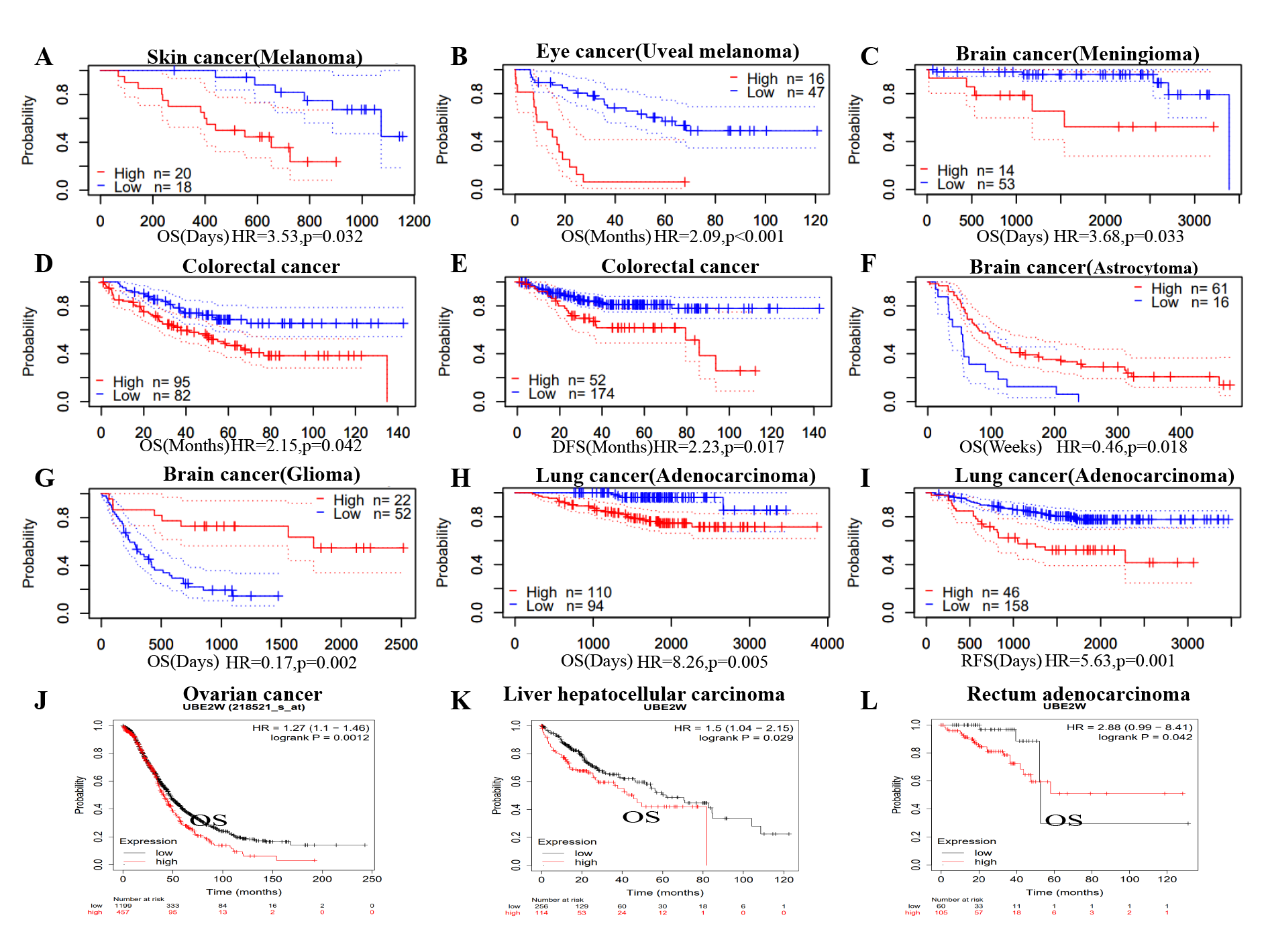


Supplementary Figure 3. (A) UBE2W protein expression profile in the HPA database. (B) Network view of theUBE2W co-expression genes in breast cancer. (C) Correlation of UBE2W expression with various genes in BRCA in TIMER. Significant positive correlations by the Spearman test are marked with red color (p <0.05). No significant correlations are marked with gray color (p>0.05).


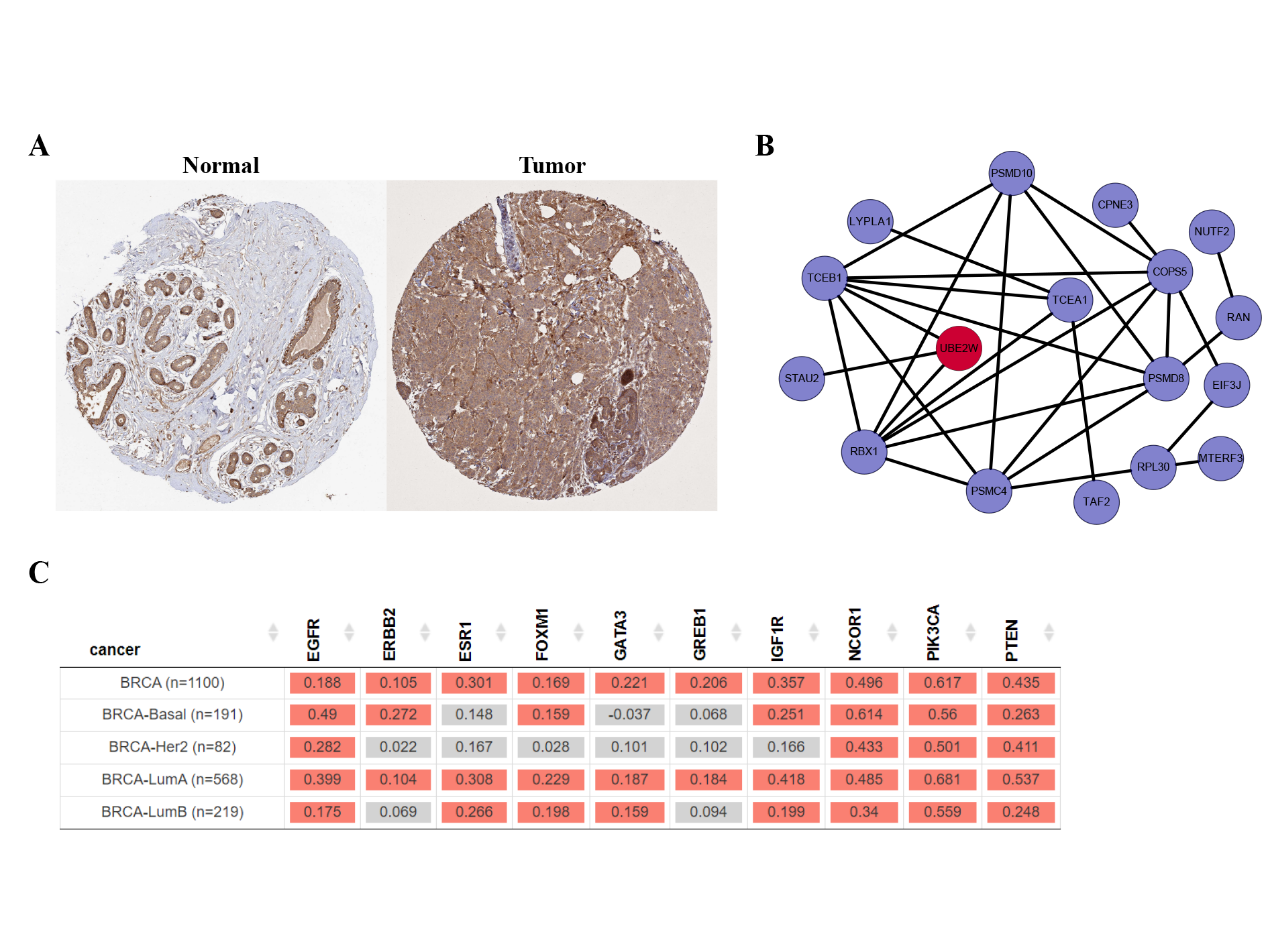


Supplementary Figure 4. Expression and prognosis value of RBX1. (A) RBX1 expression in different cancers in TIMER (***P < 0.001). (B) RBX1 expression level in BRCA in Oncomine. (C) UBE2W expression in normal, tumor, metastatic tissues in TNMplot. (E, F) Overall survival and relapse-free survival analysis of RBX1in Kaplan-Meier Plotter. (G, H) Overall survival and relapse-free survival analysis of RBX1in PrognoScan.


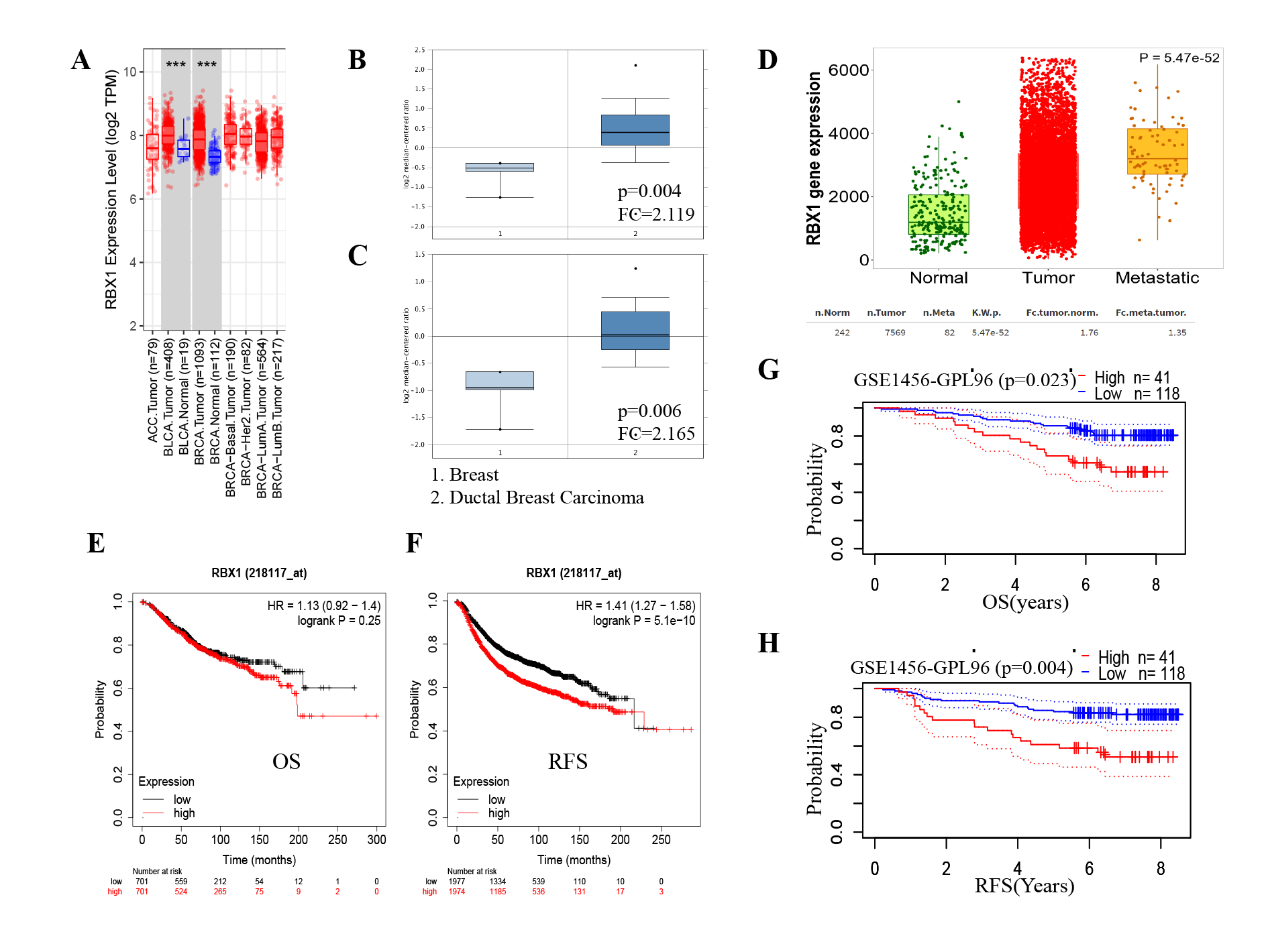


Abbreviations: FC: fold charge; OS: overall survival; RFS: relapse-free survival; HR: hazard ratio.

Supplementary Table 1. UBE2W expression in cancers vs normal tissue in Oncomine database (positive).

Supplementary Table 2. Positive results associated with UBE2W expression in different cancers from the Prognoscan database.

Abbreviations: OS: overall survival; RFS: relapse-free survival; DFS: disease-free survival; DMFS: distant metastasis free survival; NSCLC: Non-small cell lung cancer.

Supplementary Table 3. Correlation analysis between UBE2W and markers of infiltrating immune cells in TIMER.

Supplementary Table 4. Correlation analysis between UBE2W and markers of infiltrating immune cells in GEPIA.
